# The prognostic role of RBP-4 and adiponectin in patients with peripheral arterial disease undergoing lower limb endovascular revascularization

**DOI:** 10.1186/s12933-021-01411-6

**Published:** 2021-11-10

**Authors:** Nikolaos P. E. Kadoglou, Emmanouil Korakas, Christos Karkos, Eirini Maratou, Ioannis Kanonidis, Panagiotis Plotas, Nikolaos Papanas, Paraskevi Moutsatsou, Ignatios Ikonomidis, Vaia Lambadiari

**Affiliations:** 1 Medical School, University of Cyprus, 215/6 Old road Lefkosias-Lemesou, CY-2029 Aglantzia, Nicosia, Cyprus; 2 2nd Cardiology Department, “Hippokration” Hospital, Aristotle University of Thessaloniki, Thessaloniki, Greece; 3 2nd Department of Internal Medicine, Research Institute and Diabetes Centre, Athens University Medical School, Attikon University General Hospital, Athens, Greece; 4 5th Department of Surgery, Aristotle University of Thessaloniki, Thessaloniki, Greece; 5 Department of Cardiology, University of Patras Medical School, Patras, Greece; 6 Diabetes Centre, Second Department of Internal Medicine, Democritus University of Thrace, 68100 Alexandroupolis, Greece; 7 Department of Clinical Biochemistry, Medical School, National and Kapodistrian University of Athens, Athens, Greece; 8 Second Cardiology Department, Attikon University Hospital, Medical School, National and Kapodistrian University of Athens, Athens, Greece

**Keywords:** RBP4, Adiponectin, Peripheral artery disease, MACE, Inflammation

## Abstract

**Background:**

RBP4 is an adipokine with an established role in atherosclerosis, while adiponectin has unique anti-inflammatory properties. We investigated the association of RBP4 and adiponectin with the presence of symptomatic peripheral artery disease (PAD) and their possible prognostic role in major adverse cardiovascular events (MACE).

**Methods:**

We enrolled 168 consecutive patients with symptomatic, established PAD, requiring revascularization by endovascular means of any or both of their lower limbs. 88 age- and sex-matched subjects with less than 2 classical cardiovascular risk factors served as controls. Clinical parameters, glycemic and lipid profile, RBP4 and adiponectin levels were assayed. The occurrence of MACE was recorded during the 6-month follow-up and patients were assigned to MACE and non-MACE subgroups.

**Results:**

The presence of symptomatic PAD was significantly correlated with age, diabetes, hsCRP, RBP4 and low adiponectin levels (p < 0.05). After adjustment for age, RBP4 (β = 0.498, p < 0.001), and adiponectin (β = –0.288, p < 0.001) levels remained as independent predictors of PAD presence in the whole study cohort. At baseline, MACE subgroup appeared with higher RBP-4 and hsCRP serum levels than non-MACE subgroup (p < 0.001), but no differences were detected for adiponectin (p = 0.758). Serum RBP4 levels remained independent predictor of MACE (β = 0.455, p < 0.001) after adjustment for traditional cardiovascular risk factors.

**Conclusions:**

High RBP4 and low adiponectin serum levels are independently associated with PAD presence. In addition, RBP4 is an independent predictor of MACE incidence in symptomatic PAD patients.

## Introduction

Peripheral artery disease (PAD) is a manifestation and a cardinal complication of systemic atherosclerosis and is characterized by progressive stenosis of the peripheral arteries [1]. Apart from the obvious reduced functional capacity, PAD patients experience high rates of cardiovascular (CVD) morbidity and mortality, especially those with chronic, low-grade inflammation, such as diabetes mellitus and obesity [2]. Revascularization of lower-limb arteries has been associated with better long-term survival and quality of life when the patency of the arteries is maintained [3]. However, the restenosis rate and the post-intervention occurrence of major adverse cardiovascular events (MACE) is still high despite the advances in revascularization techniques and the optimal pharmaceutical therapy [4]. Traditional atherosclerotic factors such as the ankle-brachial index (ABI) have not been proven efficient to identify the PAD patients at greatest risk for MACE. Limited data indicate that inflammatory biomarkers may predict the clinical outcomes of endovascular intervention in PAD [5]. However, more studies are required to establish the prognostic role of novel biomarkers in PAD patients, candidates for revascularization [6].

In recent decades, adipose tissue has been recognized as an active secretory organ through the production of various adipokines, involved in inflammation, insulin resistance and atherosclerosis processes [7]. Retinol-Binding Protein-4 (RBP4) is a relatively novel adipokine [8] which is primarily produced in the liver and in the adipose tissue to a lesser extent, and it is the main transport protein for retinol (vitamin A) in the bloodstream [9]. RBP4 levels are elevated in adiposity and shown positive association with traditional atherosclerotic factors such as dyslipidemia and insulin resistance [10, 11]. Its mediating mechanisms include the proliferation of vascular smooth muscle cells (VSMCs), the increased production of pro-inflammatory cytokines such as tumor necrosis factor-α (TNF-α), interleukins 1β,2,8,10 (IL-1β, IL-2, IL-8, IL-10) and adhesion molecules such as vascular cell adhesion molecule 1 (VCAM-1), and the impairment of glucose metabolism and adipose tissue function [9]. Nevertheless, its association with adverse cardiovascular outcomes has not been a consistent, with data in PAD patients being even more scarce [12].

Adiponectin is a 244-aminoacid long polypeptide, almost exclusively produced by adipocytes [13]. Contrary to the other adipokines, its levels are lower in obesity, and it exerts an anti-inflammatory, insulin-sensitizing and anti-atherogenic effect. Although initial in vitro studies had firmly established the cardio-protective role of adiponectin, human studies have been contradictory [14]. Low levels of adiponectin have been associated with higher incidence of PAD [15, 16], along with an increased risk of MACE in patients with symptomatic PAD [17]. On the other hand, large-scale studies and meta-analyses have documented a positive or null association between adiponectin levels and cardiac or renal disease, along with increased rates of MACE in patients with PAD [18–21]. The reasons for these discrepancies, known as “adiponectin paradox”, are yet to be elucidated.

The aim of the present study was to evaluate the relationship of serum adiponectin and RBP4 not only with PAD, but with MACE during 6-month follow-up of patients with symptomatic PAD undergoing lower limb endovascular revascularization.

## Methods

### Participants

In the present prospective, non-randomized study, we enrolled 168 consecutive patients with symptomatic, established peripheral artery disease (PAD) requiring revascularization by endovascular means (balloon angioplasty and/or stent placement) of any or both of their lower limbs. The diagnosis of severe PAD was based on ankle-brachial index (ABI < 0.80), peripheral artery stenosis > 50% documented by duplex ultrasound and angiography and symptoms of intermittent claudication, rest pain and/or skin lesions according to the TASC guidelines [22]. Ineligible patients were considered those with concurrent conditions/diseases interfering with the expression of inflammatory mediators, like active infection, wet gangrene, severe kidney or liver impairment, morbid obesity, cancer, cardiovascular ischemic events within the recent 1 month, any major surgery or severe trauma over the last 3 months and chronic inflammatory or autoimmune diseases. We also excluded patients with PAD planned for open surgery or hybrid (open surgery combined with endovascular revascularization) due to anatomical reasons. The study was approved by the local Research and Ethics committee (IRB Nr 806/23.09.2013) and patients provided a written informed consent prior to their participation.

Among subjects visiting our outpatient clinics for check-up, we identified age- and sex-matched subjects with less than 2 classical cardiovascular risk factors (hypertension, diabetes, hyperlipidemia, current smoking, family history of premature CAD) to participate in our research. All those individuals were free from any chronic cardiovascular disease (neither coronary artery disease—CAD nor PAD) based on test of myocardial ischemia and ultrasound of peripheral arteries within the last 2 years. Finally, 88 individuals agreed to participate to the present study and served as control group.

### Study design

At baseline, all participants underwent clinical examination, blood sampling and ultrasound examination of both lower-limb arteries. Besides this, all PAD patients also underwent lower-limb angiography as a part of plan procedure and they were on optimal medical therapy, including statins (LDL target < 100 mg/dl) and antiplatelet therapy prior to intervention. All percutaneous revascularization procedures were performed as previously described [23]. Iliac lesions were predominantly treated with stenting, while stent employment was provisional for superficial femoral artery, popliteal and infrapopliteal lesions.

Post-procedure, patients were put on dual antiplatelet therapy with clopidogrel (75 mg) and acetyl salicylic acid (100 mg) for 6 months. Written dietary and exercise recommendations were provided to all participants at baseline, but without further monitoring. At the end of follow-up period (6 months), clinical examination, blood sampling and lower-limb ultrasound were repeated only in PAD patients. In case of intermittent claudication relapse accompanied by ABI deterioration and ultrasound indications of restenosis (degree of stenosis ≥ 50%), the angiographic examination was repeated. Re-revascularization was conducted in those symptomatic patients with angiographically stenosis ≥ 50% found either in stents or at lesion sites of previously balloon angioplasty. The occurrence of major cardiovascular events (MACE) including acute coronary events and peripheral artery restenosis requiring re-revascularization (either endovascular or open surgery) were recorded during the 6-month follow-up based on medical records. All cases were peer-reviewed by a multi-disciplinary team (vascular surgeons and cardiologists) which decided based on medical records whether they fulfilled the MACE criteria or not. Regarding MACE incidence, PAD patients were further divided into MACE and non-MACE subgroups for analysis purposes. It is quite common in clinical practice for PAD patients to experience MACE shortly after the revascularization procedure. Moreover, we focused on the pro-inflammatory burden of our patients to get firm conclusion about the contribution of inflammatory mediators to MACE. Thereby, we considered the 6-months period long enough to record an adequate number of MACEs, associated with adipokines.

### Clinical examination

Anthropometrical parameters, like body-mass index (BMI) and waist-to-hip ratio (WHR) were calculated in all participants by a single operator. Blood pressure was measured twice, after keeping participants at a sitting position for 15 min. There was a 5-min interval between the two measurements and the mean value was estimated. Moreover, past and current medical history including medications and co-morbidities, lifestyle habits (smoking, physical activity) were documented through structure questionnaire from our previous published work [24].

Initially the diagnosis of PAD was set using the ankle-brachial index ≤ 0.9. Thereafter, ultrasonographically- or angiographically-demonstrated stenosis (≥ 75 of the cross-sectional area) was demonstrated on ultrasound or angiogram of the iliac arteries or below (Philips CX 50 ultrasound machine, Bothell, WA 98021, USA).

### Blood assays

Fasting blood samples were obtained after overnight fasting. Glycemic and lipid parameters were all assayed in an automatic enzymatic analyzer (Olympus AU560, Hamburg, Germany). The glycated haemoglobin (HbA1c) was determined by high-performance liquid chromatography (Menarini Diagnostics, Florence, Italy) only in diabetic patients. A quantity of blood samples was collected and after a centrifugation at 2500 rpm for 10 min frozen serum samples were stored (−80 °C) until analysis in the same assay. Serum adiponectin, and RBP-4 were assayed using quantikine enzyme immunoassay commercially available kits (Phoenix Pharmaceuticals, Belmont, CA, USA and Immunodiagnostik AG, Bensheim, Germany, respectively). The intra-assay CV for RBP-4 and adiponectin were 9.7% and the inter-assay CV were 5% and %, respectively. The high-sensitivity C-Reactive Protein (hsCRP) levels were measured with latex-enhanced immunonephelometry (Dade Behring, Marburg, Germany).

### Statistical analysis

Normality of distribution was assessed with Kolmogorov-Smirnov test. Results of normally distributed continuous variables were expressed as the mean value ± SD. Continuous and categorical variables were compared using the student’s t-test and chi-square test, respectively. A two-tailed p value < 0.05 was considered to be statistically significant. Pearson’s correlation coefficient was calculated to determine the strength of the association of PAD presence with continuous variables. After univariate analysis, variables showing a significant correlation with PAD entered a logistic multiple regression analysis to check for independent associations. The computer software package SPSS (version 25.0; SPSS Inc, Chicago, IL, USA) was used for statistical analysis.

## Results

### Baseline characteristics

At baseline, we observed significant differences between PAD patients and healthy controls regarding blood pressure, smoking rates, HDL, hsCRP, RBP4 and adiponectin levels (p < 0.05). The high prescription rate of statins in PAD group may explain the absence of difference in most lipid parameters between those two groups. Nevertheless, the majority of PAD patients did not achieve the LDL target. After comparison of statin-treated and statin-free participants we observed non-significant difference in their serum levels of both RBP4 and adiponectin (data not shown). We also checked the serum levels in non-diabetic participants between PAD and control groups. Serum levels of adiponectin remained significantly lower in non-diabetic PAD patients than their non-diabetic controls (8.12 ± 2.97 vs. 11.88 ± 3.05 mg/L; p = 0.004). Non-significant difference we observed for RBP4 between non-diabetic subgroups. The clinical and laboratory characteristics of PAD and control groups are presented in Table 1.

**Table 1:** Clinical and laboratory characteristics of patients with peripheral artery disease (PAD group) and controls (CO group) at baseline

	PAD group (n=168)	CO group (n=74)	P value
Age (y)	76 ± 11	70 ± 10	0.223
Males, n (%)	141 (83.9)	60 (81)	0.929
Smoking, n (%)	43 (25.6)	13 (17.6)	< 0.001
Hypertension, n (%)	125 (74.4)	13 (17.6)	< 0.001
Dyslipidemia, n (%)	162 (96.4)	35 (47.3)	< 0.001
Statins, n (%)	160 (95.2)	19 (25.7)	< 0.001
ABI	0.48 (0.09)	1.1 (0.1)	< 0.001
Diabetes, n (%)	68 (36.9)	7 (9.5)	< 0.001
BMI (kg/m^2^)	27.98 ± 3.22	26.9 ± 4.32	0.212
SBP (mmHg)	139 ± 21	125 ± 12	< 0.001
DBP (mmHg)	85 ± 9	78 ± 8	0.405
Creatinine (mg/dl)	1.61 ± 0.4	1.1 ± 0.3	0.112
TChol (mg/dl)	183 ± 54	162 ± 41	0.105
HDL-C (mg/dl)	41 ± 8	49 ± 13	0.013
LDL-C (mg/dl)	117 ± 43	92 ± 22	0.072
TG (mg/dl)	122 ± 64	106 ± 33	0.199
FPG (mg/dl)	145 ± 35	100 ± 17	< 0.001
HbA1c (%)^a^	7.3 ± 1.4	–	–
WBC (cells/µL)	10,984 ± 2998	6234 ± 2109	< 0.001
hsCRP (mg/L)	7.64 ± 2.26	1.12 ± 0.42	< 0.001
RBP-4 (mg/L)	43.33 ± 18.88	15.11 ± 4.45	< 0.001
Adiponectin (mg/L)	6.53 ± 2.45	12.33 ± 2.1	< 0.001

Data are expressed as means ± SD. n, number of patients; BMI, body-mass index; SBP, systolic blood pressure, DBP, diastolic blood pressure; TChol, total cholesterol; TG, triglycerides; FPG, fasting plasma glucose; WBC, white blood cells; hsCRP, high-sensitivity C-Reactive Protein

^a^HbA1c was measured only in diabetic patients

### Major adverse cardiovascular events (MACE)

During the 6-months follow-up period, we recorded 69 MACE in 52 PAD patients (MACE subgroup). In particular, 8 PAD patients experienced an acute cardiovascular event (2 of them died) and another 44 PAD patients underwent at least one new revascularization procedure (endovascular or open surgery) due to restenosis, while 2 of them died due to surgical complications. The rest of PAD patients had an uncomplicated post-intervention 6-months period (116 patients: non-MACE subgroup). Therefore, 164 PAD patients completed the study.

### MACE vs. non-MACE subgroup

At baseline, MACE subgroup appeared with marginally significant elevation in LDL-C and significantly higher smoking rate, RBP-4 and hsCRP serum levels than non-MACE subgroup (p < 0.001). Those subgroups did not differ at baseline in the rest of variables, including adiponectin (p < 0.001) (Table 2).

**Table 2:** Clinical and laboratory characteristics within peripheral artery disease group at baseline regarding the MACE incidence during follow-up

	MACE subgroup (n=52)	Non-MACE subgroup (n=116)	P value
Age (y)	79 ± 13	74 ± 10	0.319
Males, n (%)	42 (80.8)	99 (85.3)	0.902
Smoking, n (%)	27 (51.9)	16 (13.8)	< 0.001
Hypertension, n (%)	42 (80.8)	83 (71.6)	0.248
Dyslipidemia, n (%)	50 (96.1)	112 (96.6)	0.991
Statins, n (%)	49 (94.2)	111 (95.7)	0.887
ABI	0.46 (0.08)	0.49 (0.09)	0.821
Diabetes, n (%)	22 (42.3)	46 (39.7)	0.947
BMI (kg/m^2^)	28.22 ± 3.35	27.87 ± 3.11	0.878
SBP (mmHg)	141 ± 23	138 ± 22	0.906
DBP (mmHg)	85 ± 8	85 ± 9	0.998
TChol (mg/dl)	196 ± 57	177 ± 40	0.255
HDL-C (mg/dl)	39 ± 7	42 ± 10	0.623
LDL-C (mg/dl)	130 ± 43	112 ± 22	0.054
TG (mg/dl)	136 ± 64	115 ± 48	0.136
FPG (mg/dl)	151 ± 40	142 ± 33	0.888
HbA1c (%)^a^	7.5 ± 1.5	7.2 ± 1.3	0.659
WBC (cells/µL)	12,110 ± 3356	10,479±2777	0.384
hsCRP (mg/L)	10.56 ± 2.92	6.33±1.85	< 0.001
RBP-4 (mg/L)	59.97 ± 21.27	35.87 ± 15.22	< 0.001
Adiponectin (mg/L)	7.22 ± 2.1	7.67 ± 2.88	0.758

Data are expressed as means ± SD. n, number of patients; BMI, body-mass index; SBP, systolic blood pressure, DBP, diastolic blood pressure; TChol, total cholesterol; TG, triglycerides; FPG, fasting plasma glucose; WBC, white blood cells; hsCRP, high-sensitivity C-Reactive Protein

^a^HbA1c was measured only in diabetic patients

### Follow-up results

Among them, we did not detect any significant change from baseline to the end of study in all clinical and laboratory parameters, while the pharmaceutical regimens remained unaltered in both subgroups (data not shown). Despite, the endovascular procedure only a slight minority of PAD smokers ceased smoking during the follow-up.

### Correlations

In the whole study cohort, the presence of symptomatic PAD was significantly correlated with age, diabetes, hsCRP, RBP4 and low adiponectin levels (p < 0.05). The latter variables entered a multiple logistic regression analysis. After adjustment for age, RBP4 (β = 0.498, p < 0.001), and adiponectin (β = −0.288, p < 0.001) levels remained as independent predictors of PAD presence (R^2^= 0.422, p < 0.001) (Table 3).

**Table 3:** Standard multiple regression analysis of PAD presence (dependent variable) and other independent variables within the whole study cohort (PAD patients and controls)

	PAD		
	β	95% CI	P value
RBP4	0.498	0.328–0.661	< 0.001
Adiponectin	−0.288	−0.363–−0.205	< 0.001
Age	0.598	0.101–1.098	0.469
Diabetes	0.083	−0.012–0.178	0.664
hsCRP	0.606	0.198–0.998	0.089

CI, confidence interval; hsCRP, high-sensitivity C-Reactive Protein

**Fig. 1:**
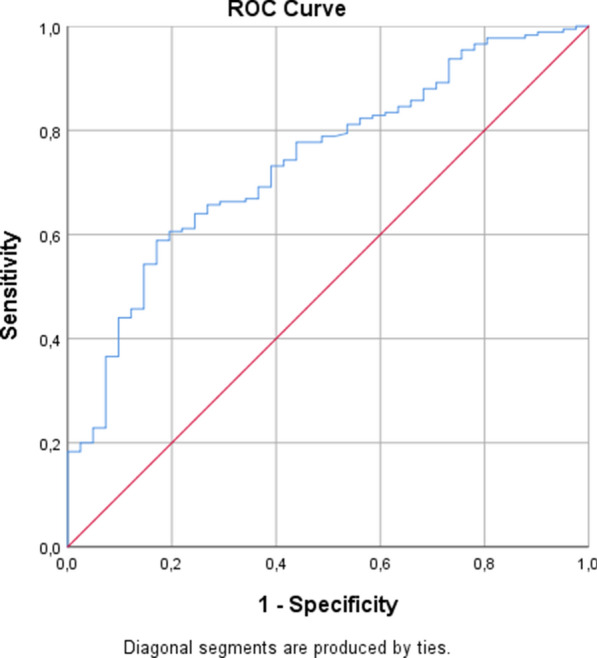
Receiver operating characteristic curve analysis of Retinol-Binding-Protein-4 (RBP4) as prognostic marker of major adverse cardiovascular events (MACE) including acute coronary events and peripheral artery restenosis requiring re-revascularization in patients with peripheral artery disease (PAD) undergoing endovascular revascularization

Similarly, we searched for univariate correlations of MACE with other variables within PAD group. Among variables, RBP4, hsCRP and smoking were significantly correlated with MACE (p < 0.05). Multiple logistic regression analysis was performed to estimate the association of MACE incidence with clinical and biochemical variables, after adjustment for traditional cardiovascular risk factors like diabetes, hypertension, dyslipidemia, smoking and male gender. Serum RBP4 levels remained independent predictor of MACE (β = 0.455, p < 0.001). Based on serum RBP4 levels as an independent predictor of MACE incidence, the area under the ROC curve in our PAD patients was 0.740 (95% CI 0.661−0.819) (Fig. 1).

## Discussion

Adipokines have been extensively studied and have been associated with cardiovascular complications. The present prospective study demonstrated higher RBP4 and lower adiponectin serum levels in patients with PAD undergoing endovascular revascularization than age- and sex-matched individuals without cardiovascular disease. During a 6-month, post-procedure follow-up, we demonstrated for first time the independent association of circulating RBP4 levels with MACE incidence in PAD patients.

### RBP4 and cardiovascular disease

Although there is a growing number of studies demonstrating the relationship of RBP4 with either coronary [25] or carotid atherosclerosis [26] to our knowledge, no previous studies have focused on patients with PAD. Our study demonstrated a positive association between RBP4 serum levels and the presence of symptomatic PAD in a cohort of PAD patients and PAD-free controls. The most striking finding of our study was the association of baseline RBP4 serum levels with the post-procedure incidence of acute coronary events or peripheral artery restenosis requiring re-revascularization. Raising a prognostic factor in PAD patients undergoing endovascular revascularization is of great clinical importance, regarding the high occurrence of restenosis and the frequent adverse cardiac events among those patients. The prognostic power of RBP4 has been supported in other populations at high cardiovascular risk like those with chronic heart failure (CHF) [27] and chronic kidney disease (CKD) [28]. Sun et al. [29] showed that full-length and total RBP4 were significant risk factors for the occurrence of coronary artery disease (CAD) among 468 women in the Nurses’ Health Study cohort, especially in the first 8 years of follow-up. On the contrary, other studies have failed to demonstrate the role of RBP4 as a predictor of ischemic stroke [30], or CAD [31]. Surprisingly, Patterson et al. [32] showed that higher RBP4 levels were associated with reduced non-CVD mortality during an average of 15.4 years follow-up.The reasons behind those discrepancies have not yet been elucidated. RBP4 regulates macrophage foam cell formation and enhances chronic endothelial inflammation through the activation of macrophages, mediated through the c-Jun-N-terminal protein kinase (JNK) and Toll-like receptor 4 (TLR-4) pathways, along with the activation of NADPH oxidase and NF-Κb [6, 33]. Furthermore, most but not all studies indicate that RBP4 induces the expression of pro-inflammatory molecules such as IL-1β, IL-2, IL-10, VCAM-1, E-selectin, TNF-α and CRP [12, 34]. In fact, RBP4 has been associated with a number of components of metabolic syndrome such as hypertension and hypertriglyceridemia independently of kidney function, such as in our cohort, while it may adversely affect the metabolic profile by increasing insulin resistance through mechanisms such as increased hepatic gluconeogenesis decreased GLUT4 translocation and impaired insulin signalling in adipose tissue [35]. In contrast, there are other data against the pro-atherogenic, pro-inflammatory effects of RBP4. Takebayashi et al. [11] showed a major role of RBP4 in increased nitric oxide (NO) production via stimulation of the PI3K/Akt/eNOS pathway and inhibition of the insulin-mediated endothelin 1 (ET-1) production, collectively leading to vasodilatation. Therefore, the exact actions of RBP4 in atherosclerosis progression and vascular homeostasis are still warranted. In addition, it has not been clarified which of the holo-RBP4 (bound to retinol, comprising 85% of total RBP4 levels) or apo-RBP4 is the main molecule responsible for the pro-inflammatory, dysmetabolic effects, and whether these actions are mediated through retinol or retinoic acid signaling [36]. A clear cut-off value of RBP4 for cardiovascular diseases has not yet been set [9]. The Western blotting technique is considered as the gold standard, but many ELISA (which do not differentiate between holo- and apo-RBP4) or other enzyme immunoassays have been employed in different cohorts [36]. It becomes thus evident that the lack of standardized and validated methods of quantification of serum RBP4 is a confounding factor for studies comparison. Finally, dietary regimens such as the DASH diet, the Mediterranean diet or high-protein diets have been associated with decreased RBP4 levels [6, 37] and, therefore, diet could have also confounded previous results including ours where the adherence to a health dietary pattern was not evaluated.

### Adiponectin and PAD

Adiponectin has been more extensively studied in PAD patients than RBP4. In agreement to previous published research, we found lower adiponectin levels within PAD patients and a significant, independent negative association between adiponectin and PAD presence [13, 38]. Iwashima et al. [39] have pointed out a significant association of adiponectin with ABI, implying a relation of adiponectin to disease severity. similarly, in a subgroup within the Health Professionals Follow-up Study consisting of 18.225 men followed-up for 14 years, adiponectin was associated with a 42% lower risk of PAD per SD increase, with only a small attenuation after adjustment for HDL-C, LDL-C, CRP and cystatin C [40].

The mechanisms underlying those favourable vascular effects of adiponectin are related to its various anti-inflammatory, anti-atherogenic properties. Adiponectin inhibits TLR-mediated activation of NFkB in macrophages, suppresses the expression of TNF-α, monocyte chemoattractant protein 1 (MCP-1) and adhesion molecules (VCAM-1, intercellular adhesion molecule-1-ICAM-1), and inhibits the transformation of macrophages into foam cells [11]. In addition, adiponectin exerts antioxidant actions, such as increasing phosphorylation of Akt and endothelial nitric oxide synthase (eNOS) at Ser^1177^ or decreasing the activation of NADPH-oxidases, together with insulin-sensitizing capacities, by reducing hepatic glucose output and stimulating glucose use and fatty acid oxidation in muscles, through phosphorylation of the insulin receptor and activation of AMP-activated protein kinase [41]. Above all, it diminishes the proliferation of VSMCs, a pivotal mechanism of atherosclerosis progression.

### Adiponectin and MACE: discrepancies and possible explanations

Despite the inverse relationship between adiponectin and PAD, we failed to demonstrate its association with MACE occurrence. This was not surprising, since research data are controversial regarding the possible prognostic value of adiponectin (positive, negative, neutral). Urbonaviciene et al. [17], have shown that a 1 mg/l increase in adiponectin levels was associated with a 22% decrease of MACE incidence in symptomatic PAD patients, but this effect was limited only to male participants. Dieplinger et al. [42], in a similar cohort to ours, showed a positive association of adiponectin with 5-year mortality in symptomatic PAD patients (RR:1.05 per 1 mg/l increase), with this independent association being lost only after adjustment for NT-proBNP (not measured in our study). In a large-scale prospective study where 4,274 patients with acute ischemic stroke were enrolled, elevated levels of adiponectin were associated with increased risk for MACE within 3 years of follow-up [43], and similar results were shown in patients with metabolic syndrome [44], concomitant hemodialysis [45] or hypertension [46]. In patients with type 2 diabetes, serum adiponectin was not associated with the progression of lower limb vascular calcification [47]. The most recent meta-analysis by Scarale et al. [18] demonstrated adiponectin was associated with an increase of 24% and 28% in pooled hazard ratios for all-cause and cardiovascular mortality, respectively, an effect which applied both to total and high-molecular-weight (HMW) adiponectin. On the opposite side and in agreement with our findings a recent study found no association between adiponectin levels and MACE occurrence in PAD patients undergoing superficial femoral artery (SFA) stenting [48].

The conflicting association between adiponectin and cardiovascular mortality is commonly termed as “the adiponectin paradox”. A possible explanation may lie in adiponectin resistance, characterized by hyperadiponectinemia due to the downregulation of AdipoR1 in insulin-resistant states and at the late stages of chronic diseases, [14]. Moreover, genetic alterations, like a single nucleotide polymorphism rs822354 in the ADIPOQ locus, may lead to increased adiponectin levels associated with cardiovascular mortality [49]. Alternatively, hyperadiponectinemia may not be a true causal factor for adverse events, but rather a compensatory mechanism in chronic inflammatory states, which may be eventually ineffective. Other studies have reported the unforeseen pro-inflammatory action of adiponectin [50]. Hence, the present study gives a potential clue for the role of adiponectin in PAD, but its prognostic value needs further, large-scale studies to be clarified.

We must emphasize the significant modification of classical cardiovascular risk factors in PAD patients prior to endovascular intervention. Unambiguously, the optimal pharmaceutical therapy has limited the influence of several cardiovascular risk factors on clinical outcomes. Thereby, the remarkable difference between PAD and control groups in RBP4 and adiponectin are evident despite the high-prescription rate of cardioprotective medications (e.g. anti-hypertensive), in the former group. Notably, previous studies have failed to demonstrate persistently any significant impact of statins on serum levels of RBP4 [9], while data about adiponectin are mixed. A large meta-analysis showed a significant increase in plasma adiponectin levels after statin therapy, with most statin members, but a deleterious decrease was observed with rosuvastatin [51].

### Limitations

The relatively small, patient cohort and the short follow-up are among the most common limitations of our study. Furthermore, it is unfeasible to determine whether RBP4 circulating levels as a stratification biomarker play a causative role on the cardiovascular risk or it is only an epiphenomenon. Another important limitation was that the minority of participants was diabetic, which might have influenced the net results of adipokines. It is possible to hypothesize that the presence of diabetes in all patients would have augmented the differences between subgroups. On the other hand, the majority of patients with classical cardiovascular risk factors (e.g. diabetes, dyslipidemia, hypertension etc.) were already on optimal pharmaceutical therapy. So, we cannot rule out the plausible effects of pharmaceutical agents on adipokines, leading to underestimation of its predictive power. Finally, we did not analyze the time of MACE incidence and thereby we could not perform Kaplan Meier analysis which could have added a time dependent pattern of RBP-4.On the other hand, we grouped PAD patients based on MACE, and thereby we focused entirely on the prognostic value of biomarkers on a relatively short-time follow-up period.

## Conclusions

This study revealed that higher RBP4 and lower adiponectin serum levels are independently associated with the presence of symptomatic PAD requiring endovascular revascularization. In addition, it is the first study demonstrating an independent positive association between RBP4 levels and MACE incidence in the post-intervention 6-month follow-up in those patients. On the other hand, no association was demonstrated between adiponectin and MACE in the same group. Thus, RBP4 could present as a novel, cost-effective, non-invasive biomarker which could assist in early risk stratification in patients with PAD undergoing revascularization. It could tailor a more intensive pharmaceutical therapy or even a different interventional approach in PAD patients. The clinical meaning of adiponectin serum levels is still controversial, and thereby may not apply in PAD. The role of those biomarkers as causative factors or bystanders could be verified by further large-scale studies. Such studies will establish the associations of these adipokines with cardiovascular disease and elucidate their true prognostic value regarding disease severity and cardiovascular mortality.

## Data Availability

The datasets used and/or analyzed during the current study are available from the corresponding author on reasonable request.

## Abbreviations

ABI: Ankle-brachial index
BMI: Body-mass index
CAD: Coronary artery disease
CHF: Chronic heart failure
cIMT: Carotid intima-media thickness
CKD: Chronic kidney disease
CVD: Cardiovascular
DBP: Diastolic blood pressure
eNOS: Endothelial nitric oxide synthase
FBG: fasting plasma glucose
FMD: Flow-mediated vasodilatation
HbA1c: Glycosylated hemoglobin
HDL-C: High-density lipoprotein cholesterol
HFpEF: Heart failure with preserved ejection fraction
HFrEF: Heart failure with reduced ejection fraction
HR: Hazard ratio
hsCRP: High-sensitivity C-Reactive Protein
ICAM-1: Intercellular adhesion molecule-1
IL: Interleukin
JNK: c-Jun-N-terminal protein kinase
LDL-C: Low-density lipoprotein cholesterol
MACE: Major adverse cardiovascular events
MCP-1: Monocyte chemoattractant protein 1
NFκB: Nuclear factor kappa-Β
NP: Natriuretic peptide
PAD: Peripheral artery disease
RBP4: Retinol-Binding Protein-4
RR: Relative risk
SBP: Systolic blood pressure
SFA: Superficial femoral artery
SNP: Single nucleotide polymorphism
T2DM: Type 2 diabetes mellitus
TChol: Total cholesterol
TGs: Triglycerides
TLR-4: Toll-like receptor 4
TNF-α: Tumor necrosis factor-α
VCAM-1: Vascular cell adhesion molecule 1
VLDL: Very-low-density-lipoprotein
VSMCs: Vascular smooth muscle cells
WBCs: White blood cells
WHR: Waist-to-hip ratio
